# Dopamine D2 Receptor Agonists as Modulators of VEGF‐A‐Driven Angiogenesis: Mechanisms, Clinical Evidence, and Translational Opportunities

**DOI:** 10.1096/fj.202600843R

**Published:** 2026-05-12

**Authors:** Venu Akkanapally, Aman Kumar, Inemai Ezhil, Manas Ranjan Sahu, Xue‐Feng Bai, Kamal S. Pohar, Sujit Basu

**Affiliations:** ^1^ Department of Pathology Ohio State University Columbus Ohio USA; ^2^ Pharmaceutical Sciences Shenzhen University of Advanced Technology Shenzhen Guangdong China; ^3^ Department of Urology University of Cincinnati Cincinnati Ohio USA; ^4^ Division of Medical Oncology, Department of Internal Medicine Ohio State University Columbus Ohio USA; ^5^ Comprehensive Cancer Center Ohio State University Columbus Ohio USA

## Abstract

Angiogenesis mediated by vascular endothelial growth factor A (VEGF‐A) is essential for physiological vascular remodeling but also drives pathological processes, including tumor growth, ocular neovascularization, and inflammation. Emerging evidence has revealed that dopamine D2 receptor (DRD2) activation is a key inhibitory pathway that counterbalances VEGF‐A‐dependent endothelial activation and vascular permeability. This review integrates current mechanistic insights into the effects of DRD2 agonists on endothelial signaling, focusing on their ability to suppress VEGF‐A‐induced proangiogenic signaling cascades. Preclinical and translational studies have demonstrated that DRD2 agonists attenuate aberrant angiogenesis, promote vascular normalization, and mitigate VEGF‐A‐induced vascular leakage in diverse pathological contexts, including malignancy, ovarian hyperstimulation syndrome, endometriosis, and inflammatory lung injury. Particular attention is given to the emerging model of tumor‐derived VEGF‐A inducing DRD2 expression within the tumor endothelium, establishing a reciprocal paracrine feedback loop with potential biomarker relevance. Finally, the clinical safety profile and pharmacologic repositioning of DRD2 agonists are evaluated, and priorities for translational research are outlined to refine dosing, scheduling, and patient selection strategies in precision antiangiogenic therapy.

## Graphical abstract

Interplay between VEGF‐A‐driven angiogenesis and DRD2‐mediated inhibition. Tumor‐derived VEGF‐A activates VEGFR2 on endothelial cells, triggering proangiogenic signaling pathways that promote abnormal vessel growth and vascular leakage. VEGF‐A also induces DRD2 expression, establishing a feedback mechanism. Activation of DRD2 by agonists suppresses VEGFR2 signaling, stabilizes endothelial function, and reduces permeability. This pathway highlights the therapeutic potential of DRD2 agonists in modulating pathological angiogenesis across multiple disease contexts.

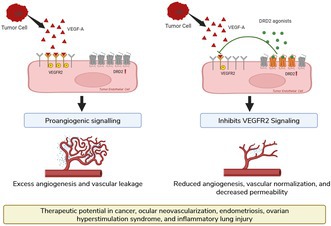

## 1 Introduction

Angiogenesis, the formation of new blood vessels from the preexisting vasculature, is a key process under both normal physiological conditions and pathological conditions such as cancer, chronic inflammation, and tissue ischemia [1, 2, 3, 4]. Dysregulated angiogenesis, which is primarily driven by vascular endothelial growth factor A (VEGF‐A), promotes tumor progression, metastasis, and treatment resistance [5]. Accordingly, the inhibition of the VEGF‐A/VEGF‐receptor 2 (VEGFR2) pathway has become a central therapeutic strategy for many solid malignancies [5, 6]. However, direct anti‐VEGF‐A therapies are limited by acquired resistance, vascular side effects, and uncertain dosing schedules, underscoring the need for alternative or adjunct strategies that can selectively restrain pathological angiogenesis while preserving physiological vessel function [5, 7, 8, 9].

Dopamine D2 receptor (DRD2) agonists represent an unexpected but increasingly compelling therapeutic approach within this context [10]. While traditionally used to treat endocrine and neurological disorders, the clinically approved DRD2 agonists bromocriptine, cabergoline, and quinagolide also exhibit vascular‐modulatory effects that intersect with VEGF‐A signaling [11, 12]. Experimental studies have revealed that the activation of DRD2 in endothelial cells inhibits VEGF‐A‐induced angiogenesis and vascular permeability [13, 14]. Beyond oncology, clinical experience in disorders such as ovarian hyperstimulation syndrome, neovascular endometriosis, and sepsis‐related lung injury supports a broader role for DRD2 agonists in reducing VEGF‐A‐driven vascular leakage without inducing the hypertension or thrombosis commonly associated with classic VEGF‐A inhibitors [15, 16, 17, 18, 19, 20] (Table 1).

**TABLE 1:** DRD2 agonists—clinical trial report.

Drug	Clinical trial	Disease	Participants	Intervention	Outcomes	Year	References
Quinagolide	Phase—4 (NCT00625950)	Endometriosis	9 Females	25–75 μg/day for 18–20 weeks	Reduction in lesion size, VEGFR2 inhibition and downregulation of pro‐angiogenic cytokines	2008	[20]
Cabergoline	Phase—3 (NCT00440258)	Ovarian hyperstimulation syndrome	60 Females	0.5 mg daily for 8 days	Reduced ascites and vascular permeability	2004–2006	[15]
Cabergoline	Phase—2 (NCT02542410)	Endometriosis	10 Females	0.5 mg twice weekly for 6 months	Reduction in endometriosis‐associated pain scores and serum VEGFR1	2016–2018	[16]

In this review, we summarize the current evidence concerning the interplay between DRD2 signaling and VEGF‐A‐mediated vascular responses. We first summarize the molecular mechanisms underlying DRD2‐mediated regulation of endothelial function and then discuss preclinical and clinical findings that highlight the therapeutic potential of DRD2 agonists for malignant and nonmalignant conditions. A particular emphasis is placed on the emerging concept of VEGF‐A‐induced DRD2 expression in the tumor endothelium, which may serve as both a biological feedback regulator and a biomarker of angiogenic activity. Finally, we explore clinical implications, safety considerations, and future research directions aimed at integrating dopaminergic modulation into precision antiangiogenic therapy.

## 2 Molecular Crosstalk Between DRD2 and VEGF‐A

DRD2 has emerged as a critical vascular regulatory node interfacing directly with the VEGF‐A signaling cascade [13, 14, 21]. Although DRD2 is traditionally recognized as a G protein‐coupled receptor that mediates neurotransmission, accumulating evidence has revealed that DRD2 also functions prominently in endothelial cells to modulate angiogenic and permeability responses [13, 14, 22]. Activation of DRD2 in endothelial and endothelial progenitor cells suppresses VEGFR‐2 phosphorylation and downstream signaling via the focal adhesion kinase (FAK) and mitogen‐activated protein kinase (MAPK) pathways [13, 14]. This inhibition impairs endothelial cell proliferation and migration—hallmarks of angiogenesis—and restricts mobilization of bone marrow‐derived endothelial progenitor cells, the rate‐limiting step in vasculogenesis, thereby markedly suppressing neovascularization [13, 14, 23].

Concurrently, DRD2 activation reduces vascular permeability by blocking the VEGF‐A‐induced phosphorylation of vascular endothelial (VE)‐cadherin, β‐catenin, and zona occludens‐1 [24]. In addition to this direct effect, DRD2 stabilizes aberrant tumor vasculature through the upregulation of angiopoietin‐1 in pericytes and Krüppel‐like factor 2 in tumor endothelial cells [25]. Together, these mechanisms enhance perfusion uniformity and may improve delivery of chemotherapeutic and immunomodulatory agents [25, 26, 27].

**FIGURE 1:**
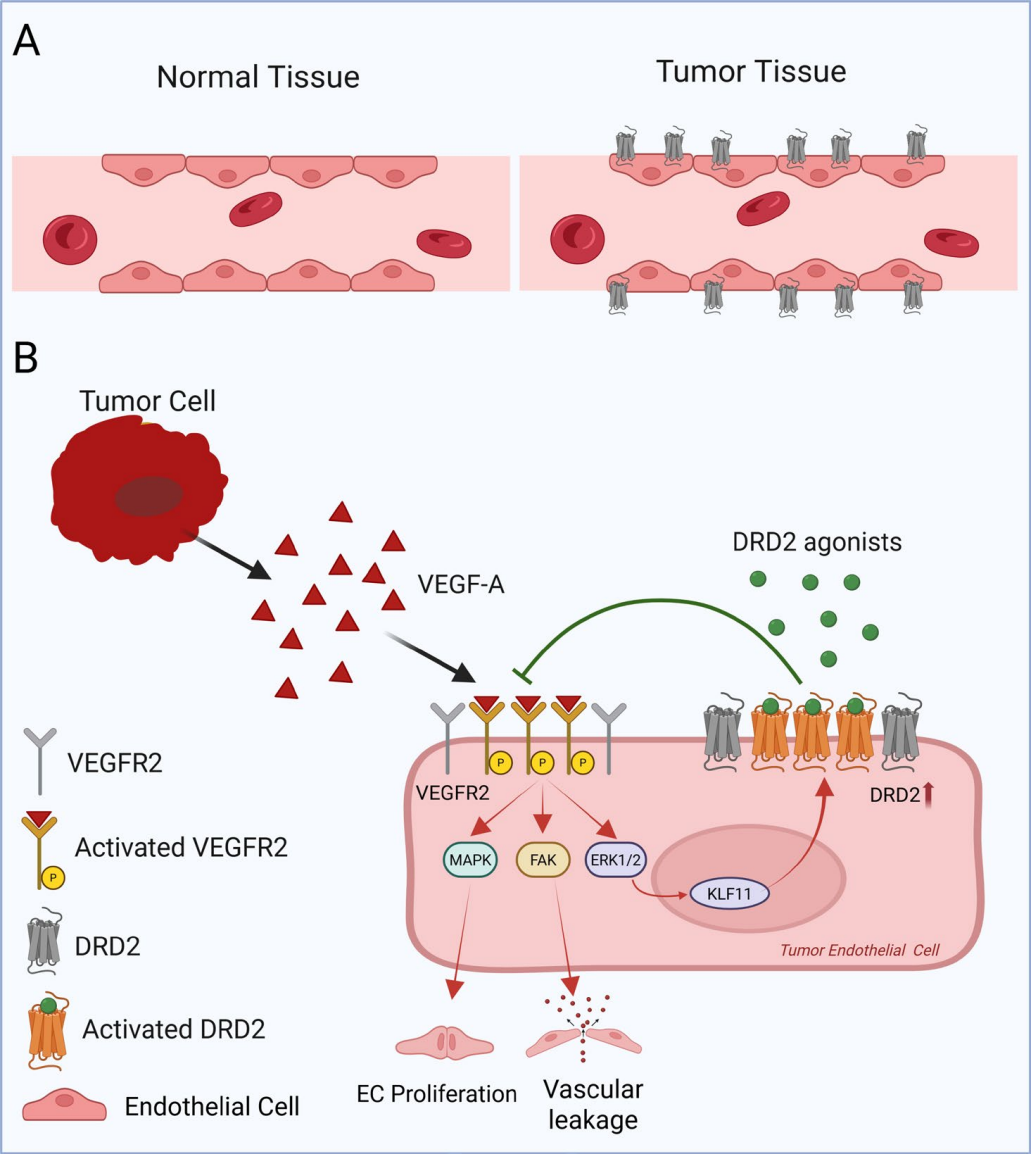
(A) Schematic comparison of endothelial DRD2 expression in normal and tumor tissues. Endothelial cells in normal tissues exhibit minimal DRD2 expression, whereas tumor‐associated endothelial cells display markedly elevated DRD2 levels. This aberrant upregulation of DRD2 in the tumor vasculature highlights its potential utility as a diagnostic marker of the tumor microenvironment and underscores its relevance as a promising therapeutic target. (B) Mechanistic illustration of crosstalk between tumor‐derived VEGF‐A signaling and DRD2 in tumor endothelial cells. Tumor cells secrete VEGF‐A, which binds to VEGFR2 on endothelial cells, leading to VEGFR2 phosphorylation and activation of downstream signaling pathways, including MAPK and FAK. Subsequently, these pathways promote endothelial cell (EC) proliferation and vascular permeabilization with concurrent upregulation of DRD2 expression in the tumor endothelial cells. However, DRD2 activation by DRD2 agonists attenuates VEGFR2 signaling outputs by normalizing the aberrant tumor vasculature and perfusion. The figure was created using BioRender.com.

Preclinical studies have further shown that tumor‐derived VEGF‐A upregulates DRD2 expression in the tumor‐associated endothelial cells through a Krüppel‐like factor 11–extracellular signal‐regulated kinase 1/2 (ERK1/2) signaling axis [21]. This induction establishes a paracrine feedback loop in which tumor‐derived VEGF‐A drives angiogenesis while simultaneously upregulating its own negative regulator, DRD2, within the tumor endothelium [21] (Figure 1).

This unique spatial and temporal regulation of DRD2 expression in the tumor microenvironment suggests its role as both an effector and a sensor of angiogenic activity. Because DRD2 expression increases upon VEGF‐A exposure [21], assessing endothelial DRD2 expression could reveal the degree of VEGF‐A pathway activation and, by extension, the angiogenic state of the tumor. Thus, DRD2 agonists can be used not only as therapeutic modulators but also as functional probes to gauge VEGF‐A dependency, representing a novel precision tool for timing antiangiogenic interventions.

Mechanistically, this evidence supports a model in which VEGF‐A and DRD2 form a tightly coupled regulatory circuit: VEGF‐A induces endothelial DRD2 expression, whereas DRD2 agonists, in turn, attenuate VEGF‐A signaling output [21]. This bidirectional communication highlights a broader principle of neurovascular crosstalk in which neurotransmitter systems contribute to fine‐tuning angiogenesis in health and disease [21].

## 3 Preclinical Evidence for DRD2‐Mediated Modulation of Tumor Angiogenesis

**FIGURE 2:**
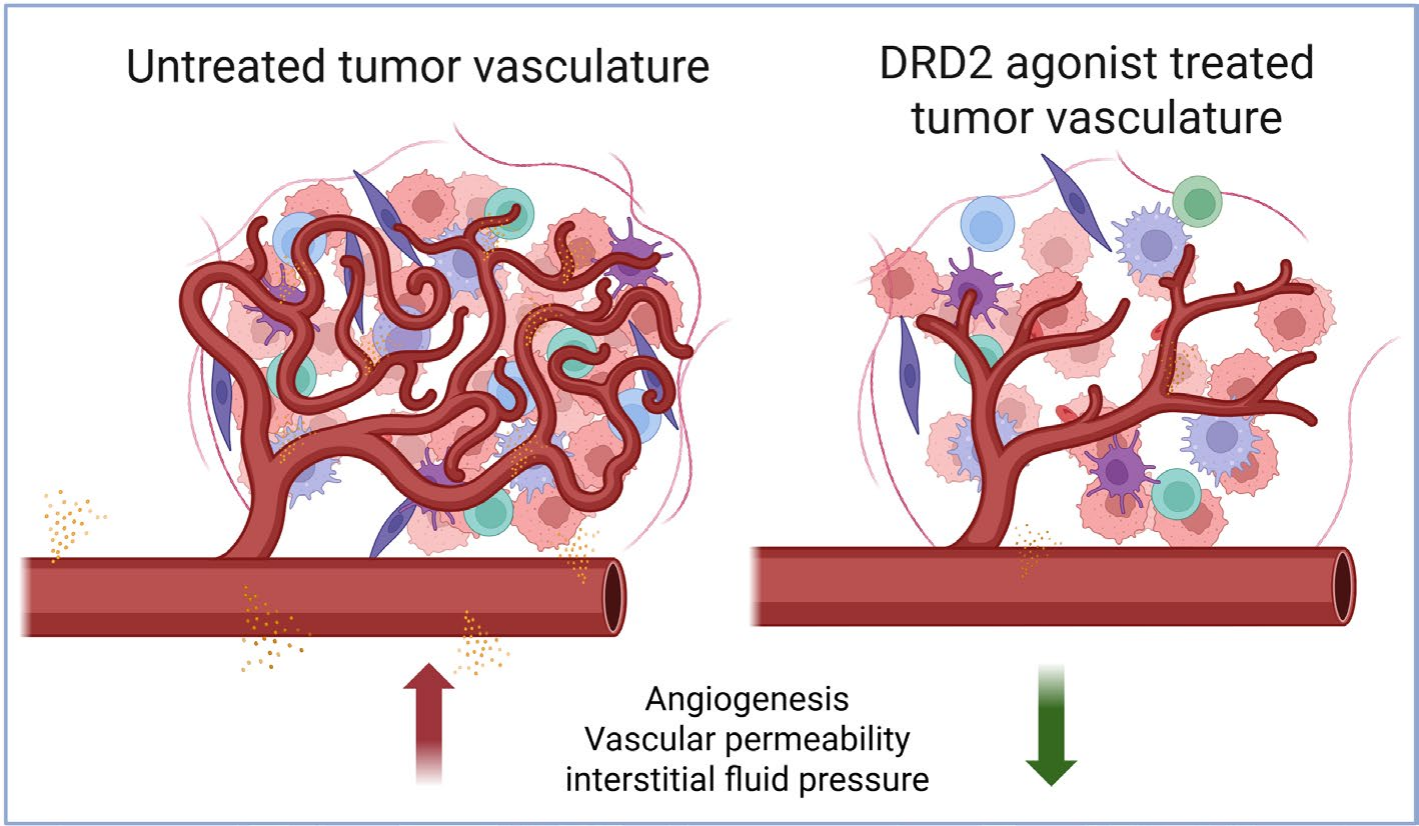
DRD2 agonist treatment normalizes tumor vasculature. In untreated tumors, the vasculature is disorganized, highly tortuous, and leaky, leading to increased angiogenesis, elevated vascular permeability, and high interstitial fluid pressure. These abnormalities contribute to poor perfusion and a hostile tumor microenvironment. In contrast, DRD2 agonist treatment promotes vascular normalization, characterized by more organized and less permeable blood vessels, reduced angiogenesis, and decreased interstitial fluid pressure. These structural and functional changes indicate suppression of abnormal angiogenesis and enhanced vascular integrity following DRD2 activation that results in suppression of tumor growth. The figure was created using BioRender.com.

Preclinical investigations of multiple tumor models have established a consistent antiangiogenic role for DRD2 activation within the tumor microenvironment. Experimental studies using murine models of lung, breast, colon, gastric, and ovarian cancers—types in which anti‐VEGF‐A agents have been clinically approved—have demonstrated that dopamine, through its selective activation of DRD2, inhibits VEGF‐A‐induced angiogenesis and vascular permeability. Consequently, dopamine [28, 29, 30] or DRD2 agonists [21, 31] have been demonstrated to markedly reduce tumor microvessel density (i.e., angiogenesis) and suppress tumor growth (Figure 2) (Table 2). These effects are largely mediated by the inhibition of VEGFR‐2 activation and downstream FAK and MAPK signaling in tumor‐associated endothelial cells [28, 29]. Importantly, compared with conventional VEGF‐A inhibitors, DRD2 activation does not appear to impair the vasculature in normal tissues [25], highlighting a degree of spatial selectivity that could translate into an improved therapeutic index. Although promising, these preclinical results necessitate clinical validation to determine the safety and therapeutic window of DRD2 agonists in the context of antiangiogenic therapy in human cancer patients.

**TABLE 2:** DRD2 agonists—pre‐clinical report.

Drug	Experimental model	Disease	Outcomes	Year	References
Dopamine^a^	Mouse tumor model	Breast and colon cancer	Reduced angiogenesis, reduced tumor growth and improved chemotherapy efficacy	2008	[28]
Dopamine^a^	Mouse tumor model	Ovarian carcinoma	Reduced microvessel density and tumor angiogenesis	2013	[30]
Cabergoline/Quinpirole	Xenograft and patient derived model	Small cell lung cancer	Reduced tumor angiogenesis and improved chemosensitization	2025	[31]

^a^ Eticlopride, a DRD2‐specific antagonist, in combination with dopamine, abrogated the anti‐angiogenic effect of dopamine, confirming that these responses are specifically mediated through DRD2 signaling.

## 4 DRD2‐Mediated Normalization of Tumor Blood Vessels

In addition to directly inhibiting vessel sprouting, DRD2 agonism has been linked to the functional normalization of the tumor vasculature [25]. Studies have shown that treatment with dopamine through its DRD2 can restore pericyte coverage, reduce vascular leakage, and improve perfusion uniformity [25] (Figure 2). These changes collectively decrease tumor hypoxia and increase the delivery of cytotoxic agents [25]. For example, in murine models, chemotherapy administered after DRD2 activation improved drug penetration into tumor tissue, resulting in superior tumor regression compared with chemotherapy alone [25]. These observations suggest that dopaminergic modulation could complement current antiangiogenic therapies by converting poorly perfused, hypoxic tumors into more drug‐accessible states [25].

Additional experimental data extend these findings to early‐stage carcinogenesis. In ultraviolet‐B‐induced cutaneous premalignancy models, DRD2 agonists suppressed angiogenesis and subsequent lesion progression [32], further linking DRD2 activation to the prevention of VEGF‐A‐driven neovascularization in diverse tumor contexts. In xenograft systems, DRD2‐selective agonists reduced ascites formation and vascular hyperpermeability, effects that parallel those observed in VEGF‐A‐dependent benign disorders [13, 14].

Collectively, these preclinical findings suggest that dopamine [13, 25, 28, 29, 30] or its D2 receptor‐selective agonists [13, 14, 21, 25, 31] act as dual‐function vascular regulators: they inhibit pathological neovascularization while reestablishing vascular homeostasis. The resulting normalization of the tumor vasculature provides a mechanistic rationale for combining DRD2‐targeted therapy with chemotherapy or immunotherapy to improve efficacy and reduce toxicity associated with potent VEGF‐A blockade [25].

## 5 Clinical Evidence Beyond Oncology

While the initial discovery of DRD2‐mediated antiangiogenic mechanisms arose from preclinical tumor models [13, 14, 21, 25, 28, 29, 30, 31], accumulating clinical evidence indicates that the dopaminergic modulation of vascular function extends well beyond oncology. Dopamine [18] and its D2 receptor‐specific agonists [15, 16] have produced beneficial vascular effects in several nonmalignant conditions characterized by pathological VEGF‐A activity. These real‐world observations provide valuable clinical validation of the preclinical findings and highlight the potential for cross‐indication therapeutic strategies.

One of the most compelling examples is ovarian hyperstimulation syndrome (OHSS), a condition driven by VEGF‐A‐induced vascular permeability that leads to ascites and edema [15]. Clinical trials have shown that cabergoline, a DRD2 agonist used for hyperprolactinemia, significantly reduces VEGF‐A‐mediated vascular leakage in patients with OHSS without affecting fertility outcomes [15]. Similar results have been reported in patients with neovascular endometriosis, where DRD2 agonists decrease lesion vascularization and inflammatory exudates, which is consistent with histological evidence of the suppression of the VEGF‐A pathway in affected tissues [16, 33].

**FIGURE 3:**
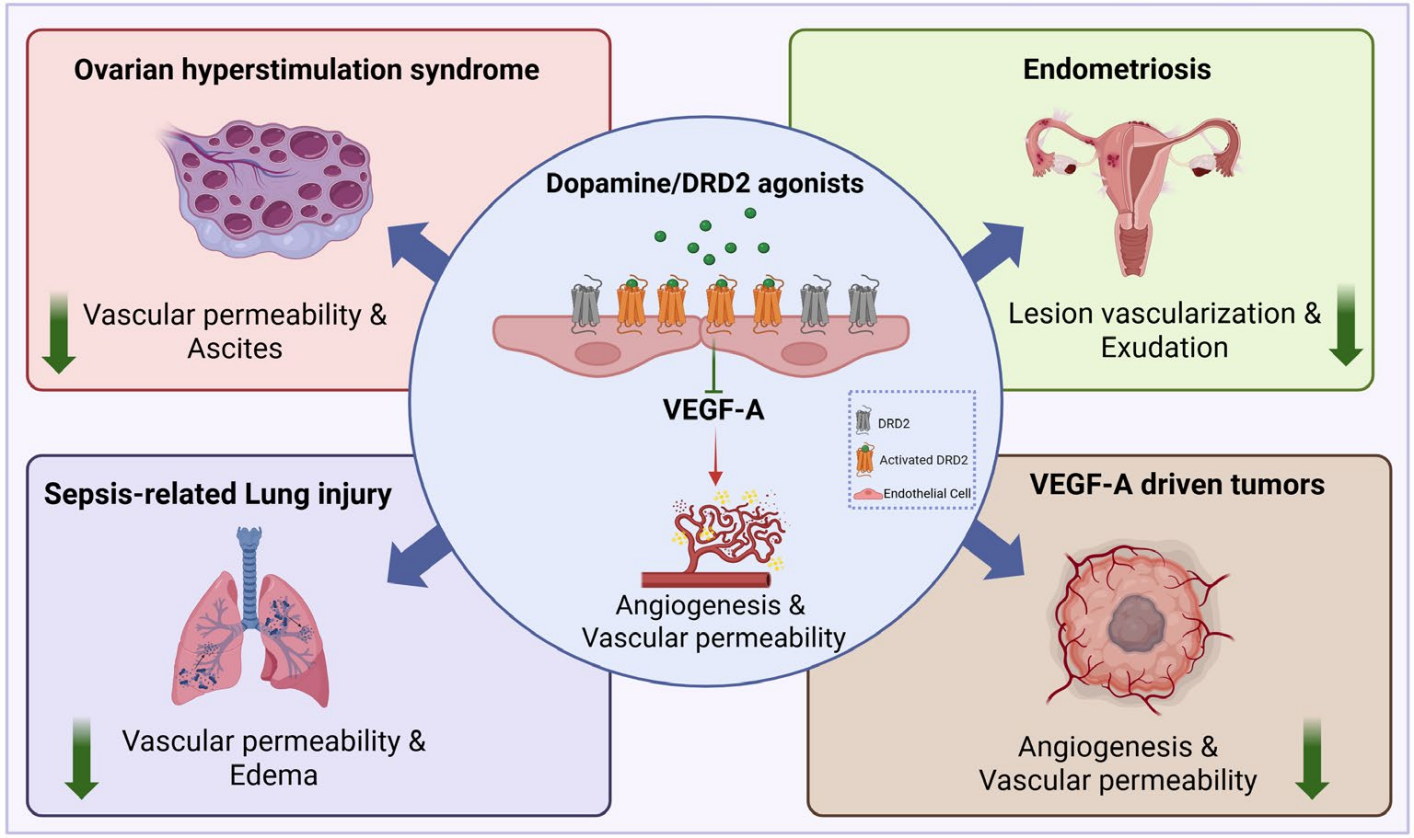
DRD2 agonists beyond oncology: Clinical evidence for non‐oncologic therapeutic potential. Beyond their ability to reduce angiogenesis and vascular permeability in VEGF‐A‐driven tumors, DRD2 agonists demonstrate therapeutic benefit across multiple non‐oncologic conditions. In ovarian hyperstimulation syndrome (OHSS), DRD2 agonists significantly reduce VEGF‐A‐mediated vascular leakage, thereby preventing ascites. In endometriosis, DRD2 agonist treatment decreases lesion vascularization and inflammatory exudate formation through inhibition of VEGF‐A signaling in affected tissues. In sepsis‐associated acute lung injury, DRD2 agonists attenuate endothelial permeability, resulting in reduced pulmonary edema and improved oxygenation. The figure was created using BioRender.com.

In critical care settings such as sepsis‐associated acute lung injury, dopamine administration has been associated with reduced pulmonary edema and enhanced oxygenation. These therapeutic effects are attributed to dopamine's capacity to inhibit VEGF‐A‐induced endothelial hyperpermeability [18]. Preclinical evidence further suggests that these protective actions are mediated through DRD2 [17]. These outcomes suggest that dopaminergic modulation maintains vascular integrity even in patients with systemic inflammatory conditions, who often present elevated VEGF‐A levels [18] (Figure 3). Additionally, translational and epidemiological data from patients with ocular neovascular diseases, including age‐related macular degeneration, indicate that DRD2 agonists may thwart pathological vessel growth and vascular permeability [34], expanding their potential application to organ‐specific pathological angiogenesis.

Across these diverse conditions, a consistent mechanism emerges: DRD2 activation counteracts VEGF‐A‐driven endothelial hyperpermeability and neovascularization without the adverse vascular events typical of direct VEGF‐A blockade, namely, hypertension, thromboembolism, or delayed wound healing [15, 16, 19]. This safety profile is particularly encouraging for oncological settings, where cardiovascular comorbidities often limit the use of antiangiogenic therapy [35]. Collectively, these clinical insights reinforce the concept that DRD2 agonists can serve as physiologic “modulators” rather than as blunt inhibitors of angiogenesis, preserving the delicate balance between neovessel suppression and the maintenance of normal vascular homeostasis.

## 6 Therapeutic Relevance and Safety Profile

The favorable safety and pharmacological profiles of DRD2 agonists make them especially attractive as adjunct or alternative antiangiogenic agents [19]. Drugs such as cabergoline and quinagolide have been in long‐term clinical use for treating hyperprolactinemia, pituitary adenomas, and Parkinson's disease, and extensive safety data are available in both acute and chronic settings [19, 33, 36]. Additionally, the ergot DRD2 agonist, cabergoline, does not show cardiac valvular defects at the low doses used to inhibit VEGF‐A‐induced angiogenesis [36]. Furthermore, unlike direct VEGF‐A inhibitors, which commonly induce hypertension, proteinuria, and thromboembolic complications [37], DRD2‐targeted agents rarely cause vascular toxicity [19, 36]. Their most frequent adverse events, such as mild nausea, orthostatic hypotension, or fatigue, stem from dopaminergic rather than endothelial mechanisms and are typically reversible or manageable with dose adjustment [19, 36].

Mechanistically, this superior safety profile likely stems from the selective activity of DRD2 agonists on VEGF‐A‐stimulated endothelial cells, where DRD2 expression is pathologically upregulated [13, 21]. Previous studies have shown minimal or absent DRD2 expression in endothelial cells of normal human lung and colon tissues, contrasting with robust expression in tumor‐associated endothelium in lung and colon cancers, where VEGF‐A levels are elevated [21, 38]. A comparable pattern has been reported in murine models [13, 21]. This differential expression enables DRD2 agonists to exert context‐dependent inhibition of VEGF‐A‐driven angiogenic signaling while sparing normal vascular homeostasis. Such tumor‐ or lesion‐focused effects may explain why clinical trials in patients with OHSS and neovascular endometriosis have shown potent anti‐VEGF‐A activity but preserved reproductive and vascular health [15, 16, 33].

In the oncologic setting, DRD2 agonists offer several potential therapeutic advantages. Because they are inexpensive, well‐tolerated, and orally available—which improves patient compliance—these agents are well‐positioned to promote health equity [19]. Additionally, these agonists provide a feasible means of sustained antiangiogenic modulation either as monotherapy in VEGF‐A‐driven tumors or as a maintenance approach following intensive VEGF‐A blockade. DRD2 activation can complement cytotoxic or immunotherapeutic regimens by normalizing the tumor vasculature, thereby improving tissue oxygenation and drug delivery [25]. Moreover, DRD2 agonists may be especially valuable for patients who are unable to tolerate conventional anti‐VEGF‐A drugs because of cardiovascular risk or long‐term toxicity concerns [35].

Together, these features align DRD2‐directed therapy with the broader goal of precision‐guided angiogenesis modulation: fine‐tuning rather than completely interrupting VEGF‐A signaling to maximize efficacy while minimizing systemic damage. By leveraging safe, clinically familiar agents [19], the DRD2 pathway provides a translationally accessible avenue to achieve this therapeutic balance.

## 7 Translational Outlook and Future Directions

The expanding recognition of DRD2 agonists as modulators of VEGF‐A‐driven angiogenesis reveals a promising translational pathway, yet significant questions remain before their widespread clinical adoption. A key priority is to systematically define DRD2 expression patterns across tumor types and disease stages. Because VEGF‐A upregulates DRD2 expression in endothelial cells [21], the presence of endothelial DRD2 could serve as a surrogate marker of VEGF‐A activity, identifying tumors that are most likely to respond to DRD2 agonist‐based therapy. Comprehensive molecular profiling of patient‐derived tissues integrating VEGF‐A levels, DRD2 expression, and vascular morphology could guide precise patient selection for future trials.

**FIGURE 4:**
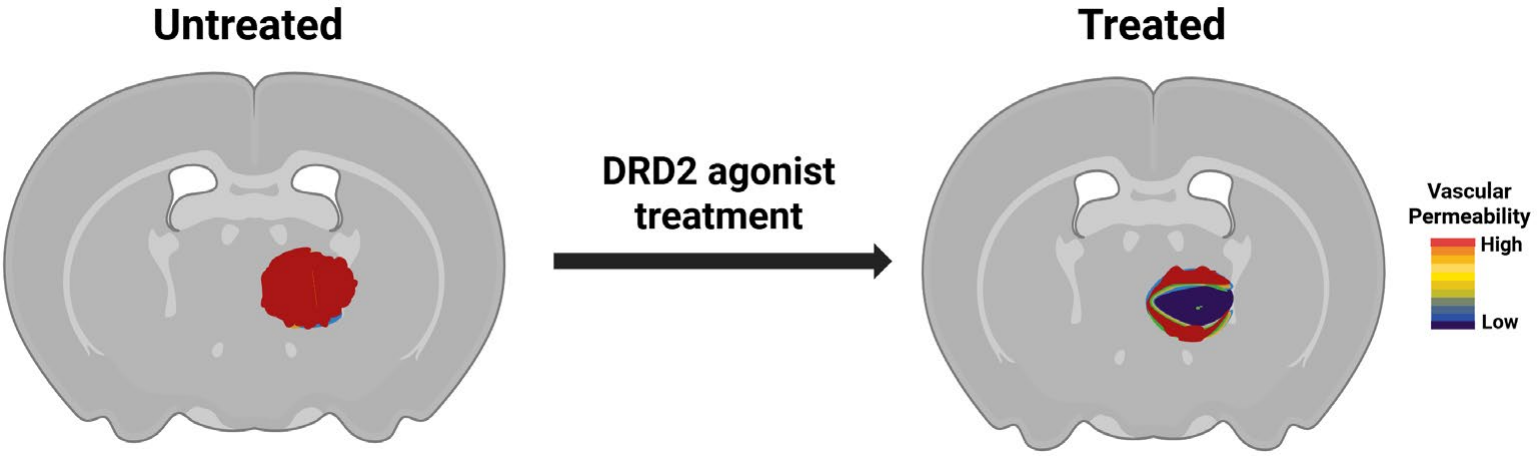
DRD2 agonist treatment decreases tumor vascular permeability. Representative dynamic contrast‐enhanced magnetic resonance imaging (DCE‐MRI) depicting vascular permeability in untreated and DRD2 agonist‐treated tumors. In the untreated group, the tumor shows high vascular permeability, reflected by extensive leakage throughout the tumor mass. Following DRD2 agonist treatment, vascular permeability is significantly reduced, demonstrated by the shift toward regions of lower permeability within the tumor. The color scale indicates relative permeability levels, with red representing high permeability and blue/purple representing low permeability. These results suggest that DRD2 activation reduces vascular leakage and promotes vascular stabilization within the tumor microenvironment, which could be useful to determine optimal timing for anti‐angiogenic therapy. The figure was created using BioRender.com.

Clinically, prospective studies will need to examine both monotherapy and combination strategies. Early‐phase trials could explore DRD2 agonists as adjuncts to standard chemotherapy, immunotherapy, or VEGF‐A blockade, focusing on the safety, pharmacodynamics, and imaging correlates of vascular normalization. We hypothesize that a transient dopaminergic challenge characterized by brief exposure to DRD2 agonists and monitored via dynamic imaging of vascular permeability can identify a window of vascular normalization. We propose that utilizing this protocol to guide the optimal timing of antiangiogenic drug administration will enhance therapeutic delivery and efficacy [39, 40] (Figure 4). This functional approach shifts the paradigm from fixed dosing schedules to adaptive regimens synchronized with real‐time angiogenic activity.

Mechanistic investigations should also address how DRD2 activation interacts with other angiogenic mediators in addition to VEGF‐A. Elucidating these networks could reveal synergistic or compensatory pathways influencing therapeutic responses.

From a pharmacological perspective, the development of DRD2 agonists with optimized endothelial selectivity and reduced central nervous system penetration could increase safety and efficacy for oncologic use. Repurposing existing formulations may accelerate progress, but rational drug design guided by receptor distribution and tumor‐specific pharmacokinetics is critical for precise targeting.

Ultimately, translating DRD2 biology into a clinical benefit will depend on integrating an understanding of its mechanism, biomarker development, and innovative trial design. If validated, DRD2 activation could evolve into a dynamic diagnostic‐therapeutic axis, providing both a modulator of pathological angiogenesis and a tool to measure VEGF‐A‐driven vascular activity in vivo [21].

## 8 Conclusions

The convergence of neuroendocrine pharmacology and vascular biology through DRD2 signaling represents an important advance in the field of angiogenesis research [11, 12]. Evidence from molecular, preclinical, and clinical studies consistently demonstrates that the activation of DRD2 counteracts VEGF‐A‐driven angiogenesis and vascular permeability while maintaining normal vascular integrity [13, 14, 21, 25, 28, 29, 30, 31]. This dual capacity to inhibit pathological neovascularization and simultaneously normalize vessel structure distinguishes DRD2 agonists from traditional anti‐VEGF‐A agents [13, 14, 25, 37] and supports their evaluation as safer, context‐specific modulators of the tumor vasculature.

The selective induction of DRD2 expression in tumor‐associated endothelial cells introduces the concept of a VEGF‐A/DRD2 feedback loop, providing both mechanistic insights and translational potential [21]. As a biomarker of angiogenic activity, endothelial DRD2 may guide patient selection and therapeutic timing. In contrast, as a pharmacologic target, its activation may optimize the efficacy and tolerability of antiangiogenic regimens.

Given their established clinical safety, oral availability, and low cost, DRD2 agonists occupy a unique position in the evolving landscape of angiogenesis‐directed therapy [19]. Future studies integrating molecular biomarkers, imaging, and adaptive treatment design will determine how best to harness these agents in oncology and other VEGF‐A‐driven diseases. Together, these developments could transform DRD2 agonists from neuroendocrine drugs into versatile, precision‐guided tools for vascular modulation in cancer and beyond.

## Author Contributions

**Venu Akkanapally:** literature search, data analysis and drafted and/or critically revised the work. **Aman Kumar:** literature search and drafted and/or critically revised the work. **Inemai Ezhil:** literature search, drafted and/or critically revised the work. **Manas Ranjan Sahu:** literature search and drafted and/or critically revised the work. **Xue‐Feng Bai:** critically reviewed the work. **Kamal S. Pohar:** critically reviewed the work. **Sujit Basu:** idea of the article, literature search, data analysis, drafted and/or critically reviewed and revised the work.

## Funding

This study was supported by HHS | NIH | National Cancer Institute (NCI), R01CA292598, R01CA285726. DOD | MHS | Congressionally Directed Medical Research Programs (CDMRP), W81XWH2110874. Ohio State University Comprehensive Cancer Center—Arthur G. James Cancer Hospital and Richard J. Solove Research Institute (The James) to Sujit Basu.

## Conflicts of Interest

The authors declare no conflicts of interest.

## Data Availability

Data sharing not applicable to this article as no datasets were generated or analyzed.
